# Supplementary material for: Fatty acyl-CoA reductase FAR1 is essential for the testicular seminolipid synthesis required for spermatogenesis and male fertility
Source: J Biol Chem. 2025 Apr 25;301(6):108538. doi: 10.1016/j.jbc.2025.108538 (PMC12149581; doi:10.1016/j.jbc.2025.108538)
Supplement: Supporting Information [file mmc1.zip › Supplementary Figures.pdf]

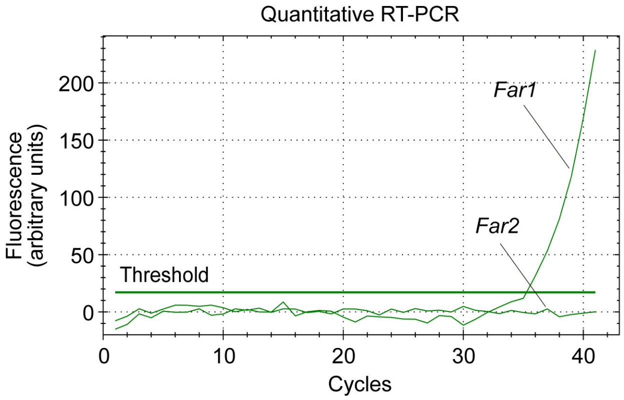

Supplementary Figure S1. Representative amplification curves from quantitative RT-PCR analysis. Total RNAs were extracted from the testes of 8-week-old male WT mice and subjected to quantitative RT-PCR using specific primers for *Far1* and *Far2*.

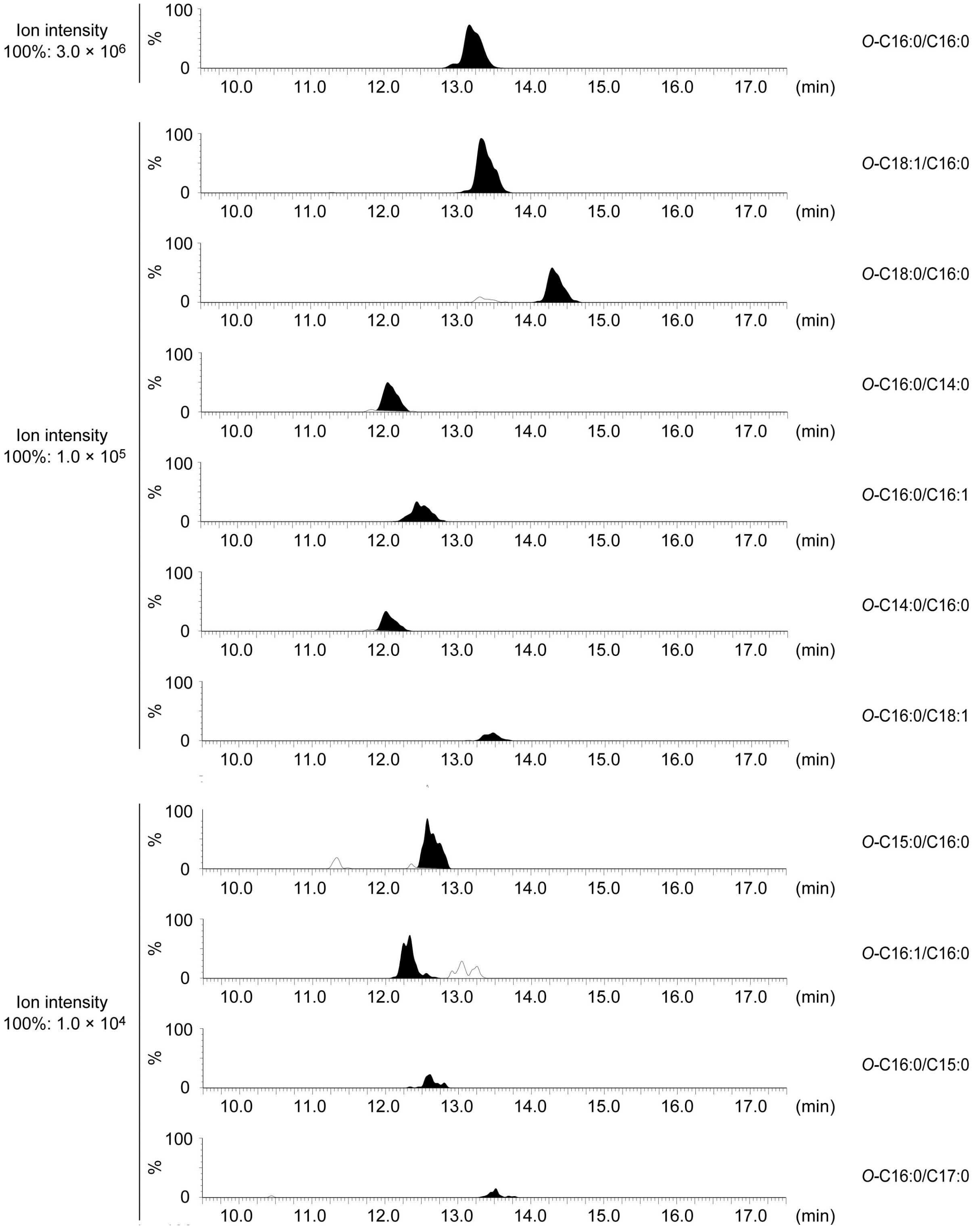

Supplementary Figure S2. Representative ion chromatograms from LC-MS/MS analysis of seminolipids. Lipids were extracted from the testes of 8-week-old C57BL/6 mice and

seminolipids were quantified via LC-MS/MS. The ion chromatograms of 11 species detected in the analysis are presented.

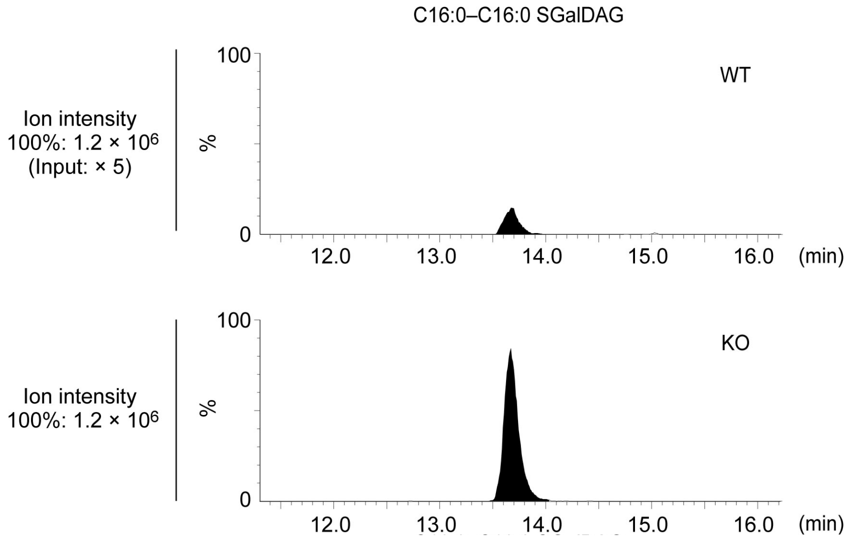

Supplementary Figure S3. Representative ion chromatograms from LC-MS/MS analysis of SGalDAGs. Lipids were extracted from the testes of 8-week-old WT and *Far1* KO mice and SGalDAGs were quantified via LC-MS/MS. Approximately 2.5-fold larger quantities of lipids from WT mice than from *Far1* KO mice were subjected to the analysis. The ion chromatograms of C16:0–C16:0 species are presented.

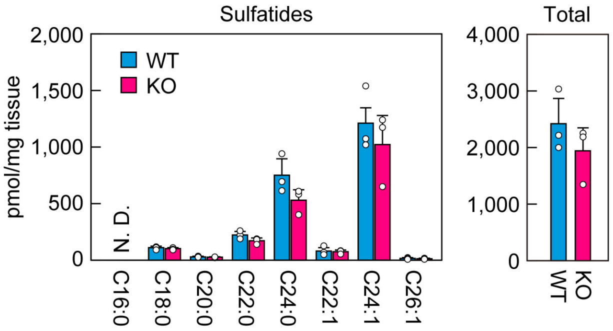

Supplementary Figure S4. LC-MS/MS analysis of sulfatides in the brain. Lipids were extracted from the brains of 8-week-old WT and *Far1* KO mice and the non-hydroxy forms of sulfatides were quantified via LC-MS/MS. Values presented are means + SD of sulfatide species containing the indicated acyl moiety (left) and their total quantity (right) (n = 3). N. D., not detected.
